# Voucher for Healthy Foods and Diabetes Control: A Randomized Clinical Trial

**DOI:** 10.1001/jamainternmed.2025.5420

**Published:** 2025-10-20

**Authors:** Nav Persaud, Moizza Zia Ul Haq, Adelaide Buadu, Areesha Sabir, Lavanya Sinha, Kevin E. Thorpe, Keying Xu, Stephen W. Hwang, Andrew D. Pinto, Enza Gucciardi

**Affiliations:** 1 Department of Family and Community Medicine, University of Toronto, Toronto, Ontario, Canada; 2 MAP Centre for Urban Health Solutions, Li Ka Shing Knowledge Institute, St Michael’s Hospital, Unity Health Toronto, Toronto, Ontario, Canada; 3 Department of Family and Community Medicine, St Michael’s Hospital, Unity Health Toronto, Ontario, Canada; 4 Dalla Lanna School of Public Health, University of Toronto, Toronto, Ontario, Canada; 5 Hospital for Sick Children, Toronto, Ontario, Canada; 6 Applied Health Research Centre, Li Ka Shing Knowledge Institute, St Michael’s Hospital, Unity Health Toronto, Toronto, Ontario, Canada; 7 Department of Medicine, University of Toronto, Toronto, Ontario, Canada; 8 School of Nutrition, Toronto Metropolitan University, Toronto, Ontario, Canada

## Abstract

**Importance:**

Programs to improve access to healthy foods for people with diabetes exist in some jurisdictions but not others, and their effects on disease control are not established.

**Objective:**

To measure the effect of a monthly grocery store voucher that allows access to healthy foods on diabetes control in patients experiencing food insecurity.

**Design, setting, and participants:**

This randomized parallel-arm clinical trial was conducted from March 21, 2023, to April 1, 2025, at 7 sites in Toronto, Canada. Patients were randomly allocated to a monthly voucher or usual access to healthy foods. Eligibility required hemoglobin A_1c_ (HbA_1c_) levels from 6.0% to 11% and food insecurity. Data analysis was conducted in May 2025.

**Intervention:**

A $65 monthly voucher ($85 for those in households with 6 or more individuals) for 6 months.

**Main Outcomes and Measures:**

The primary outcome was change in HbA_1c_ levels from baseline to 6 months. Secondary outcomes included levels of β-carotene and ascorbic acid, self-reported fruit and vegetable consumption, financial security, food security, and general health.

**Results:**

Of 1061 patients approached, 390 were enrolled (mean [SD] age, 60 [13] years; 191 [49%] men, 198 [51%] women); 81 (21%) identified as Black, 93 (24%) identified as South Asian, and 105 (27%) identified as White. Overall, 194 (50%) were randomly allocated to receive the voucher and 196 (50%) to control. The nonsignificant mean difference between voucher and control in the change in HbA_1c_ was −0.18% (95% CI, −0.41 to 0.05; *P* = .13; mean difference adjusted for baseline HbA_1c_, age, sex, household size, and condition, −0.10%; 95% CI, −0.33 to 0.12; *P* = .35). The voucher improved self-reported vegetable consumption (44.7% vs 21.5% 2 or more times per day), fruit consumption (42.6% vs 22.7% 2 or more times per day), food insecurity (risk difference, −0.10; 95% CI, −0.18 to −0.02), and general health (odds ratio, 1.6; 95% CI, 1.1-2.3) but not financial insecurity (risk difference, −0.07; 95% CI, −0.16 to 0.02) or β-carotene level (mean difference, 0.01 µmol/L; 95% CI, −0.08 to 0.06) and ascorbic acid level (mean difference, −0.18 µmol/L; 95% CI, −4.3 to 4.7).

**Conclusions and Relevance:**

In this randomized clinical trial, a monthly food voucher did not have an effect on HbA_1c_ control or objective measures of diet quality but the voucher did increase self-reported vegetable and fruit consumption.

**Trial Registration:**

ClinicalTrials.gov Identifier: NCT05776420

## Key Points

**Question:**

For patients with type 2 diabetes experiencing food insecurity, does a monthly grocery store voucher improve glycemic control?

**Findings:**

In this randomized clinical trial of 390 adults, a monthly voucher that allowed access to healthy foods did not improve diabetes control but did increase self-reported vegetable and fruit consumption.

**Meaning:**

Because monthly food vouchers were insufficient to improve diabetic control for people with food insecurity in this trial, further experimental data are needed to determine the health effects of interventions that improve food access.

## Teaser

This randomized clinical trial measures the effects of a monthly voucher that allows primary care patients with elevated hemoglobin A_1c_ to access healthy foods.

## Introduction

Diabetes prevalence is large and growing, and the risk of dying of diabetes is higher for people with a low income.^1^ Healthy foods that improve glycemic control cost more than foods that may worsen it, and this is one explanation for disparities in avoidable diabetes morbidity and mortality.^1,2,3,4,5^ Consuming vegetables and fruits is recommended for people with diabetes based on clinical trials and observational studies showing health benefits.^6,7,8^ On the other hand, processed food consumption is associated with poor health generally and poor control of diabetes; yet, processed foods are an example of foods commonly consumed because of their low price and the way they are marketed.^9,10,11,12^ The prevalence of food insecurity, which is defined as the lack of regular access to safe and nutritious foods, rose to 25% in 2023.^13^

Improving access to healthy foods may improve glycemic control and potentially health outcomes in some contexts, based on observational studies and some clinical trial results.^14,15,16^ Studied interventions include directly providing healthy foods such as vegetables to people, delivering medically tailored meals, and providing financial supports that allow people to purchase healthy foods.^17,18,19^ Produce prescription programs, where free or discounted vegetables and fruits are provided by health care professionals to people with conditions such as diabetes, have been shown to improve dietary intake, reduce food insecurity, and improve surrogate health markers in some studies.^20,21,22^ Policies and programs that promote access to healthy foods for people with diabetes vary between jurisdictions, with some providing financial supports intended to improve consumption of healthier foods.^15,23,24,25,26,27^

The purpose of this randomized clinical trial was to measure the effects of a monthly voucher that allows primary care patients with elevated hemoglobin A_1c_ (HbA_1c_) to access healthy foods.

## Methods

### Design

We conducted a multicenter, parallel, 2-group, superiority, participant-unblinded, outcome assessor-blinded, individual 1:1 randomized clinical trial at 7 primary care sites in Toronto, Canada, that enrolled patients between March 21, 2023, and October 3, 2024. Food insecurity has been declared an emergency in the City of Toronto in 2024, where food banks served 3.49 million client visits in just 1 year.^28,29^ The trial was registered (ClinicalTrials.gov NCT05776420) and the protocol is available (Supplement 1). We report the trial in accordance with the Consolidated Standards of Reporting Trials (CONSORT) reporting guidelines and the intervention is described using the Template for Intervention Description and Replication checklist.^30,31^ The trial was approved by the research ethics boards of Unity Health Toronto, Regent Park Community Health Centre, and South Riverdale Community Health Centre. All enrolled participants provided written informed consent.

### Trial Participants

We included adult (19 years or older) primary care patients with HbA_1c_ levels between 6.0% and 11.0% within the past 3 months who reported either food insecurity or financial insecurity. Food insecurity was assessed using 6 standard and validated items adapted from the Canadian Community Health Survey (Household Food Security Survey Module), applied to the past 6 months.^32^ Participants were considered eligible for the study if they either scored 2 or above on the 6-item food insecurity instrument (based on Health Canada’s approach to interpreting the data from the Household Food Security Survey Module^33^) or reported trouble “making ends meet.”^34^ We excluded patients with life-threatening allergies to common foods, with a blood dyscrasia that interferes with HbA_1c_ interpretation, requiring total parenteral nutrition, with a life expectancy of less than 6 months, or who live with a trial participant. We also excluded individuals with poorly controlled diabetes (HbA_1c_, >11%) as interventions such as intensification of pharmacological treatment might have a larger role than changes in diet.

The 7 trial sites were the Health Centre at 80 Bond, 61 Queen Family Practice Unit, Wellesley–St James Town Health Centre, Sumac Creek Health Centre, St Lawrence Health Centre, Regent Park Community Health Centre, and South Riverdale Community Health Centre. In Canada, primary care patients generally have publicly funded access to primary care that includes diagnostics such as blood work and advice from a dietitian, as well as care from specialists and during hospital admissions. Patients at participating sites were informed of the study through posters and handouts distributed at the primary care sites. Clinicians prepared lists of potentially eligible patients and provided them to trial personnel who contacted patients by mail, email, and phone.

### Trial Procedures

Study data were collected and managed using REDCap electronic data capture tools hosted at Unity Health Toronto.^35,36^ Race and ethnicity data were collected in-person or over the phone at the enrollment visit. Participants were told by a research assistant, “You may belong to one or more racial or cultural groups on the following list,” and provided with a list of possible groups with which they might identify. Participants were given the option to select “other” and describe their racial or cultural group in their own words. Participants were also given the option to select “do not know” or to decline to answer. Randomization was stratified by condition: “prediabetes” (HbA_1c_, 6.0%-6.4%) vs diabetes (HbA_1c_, ≥6.5%) and administrative site. Randomization and allocation concealment were achieved through a central web-based tool that was accessed through the REDCap electronic case report forms application, stratified by site and condition, and using permuted blocks of 2 to 4. Participants and clinicians were unblinded to allocation, whereas outcome assessors and adjudicators were blinded to allocation.

Patients randomized to the intervention group were mailed a reloadable voucher in the form of a gift card for a commercial supermarket chain of their choice. Each month for 6 months, the balance of the voucher was reloaded by $65, or $85 for participants who lived in a household of 6 or more individuals. Intervention participants were instructed by study personnel to use the cards only toward the purchase of fresh or frozen fruits and vegetables, and they received a brochure outlining the benefits of healthy eating for patients with diabetes, as well as tips and other useful information to help manage diabetes. Although participants were asked to use vouchers only for fruits and vegetables, the vouchers could be used to purchase anything sold by the supermarket. All patients had access to primary care clinicians and dietitians who provided advice about healthy eating through individual sessions, group sessions, and resources such as handouts, and all participants still had access to their other regular food sources.

### Outcomes

The follow-up period was 6 months. The prespecified primary outcome was the change in HbA_1c_ from baseline. The prespecified secondary outcomes were serum β-carotene level, serum ascorbic acid (vitamin C) level, patient-reported fruit and vegetable consumption (measured by the frequency of daily fruit and vegetable consumption using a question from the Canadian Community Health Survey),^37^ patient-reported food security, patient-reported financial security, and patient-reported general health. We plan to separately report the prespecified secondary outcome of health care utilization once health administrative data are available.

### Sample Size Calculation

The sample size was estimated to detect a 0.4% difference between groups in the change in HbA_1c_ levels. We assumed a mean (SD) change of 0% (2%) in the control group and a correlation of baseline and follow-up results of 0.75. For 80% power and an α of .05, 173 were required in each group. To account for a potential 10% rate of missing data or participant drop-outs, our objective was to recruit 195 individuals in each group, resulting in a total target of 390 participants across both groups.

### Statistical Analysis

We used an intention-to-treat analysis. For the difference between groups in the change in HbA_1c_, we analyzed 6-month HbA_1c_ by analysis of covariance, both adjusted for only baseline HbA_1c_ and adjusted for baseline HbA_1c_, age, sex, household size, and condition (diabetes vs prediabetes). The proportions of changing condition status were compared using Fisher exact tests. For the exploratory subgroup analyses of the difference between groups in the change in HbA_1c_ (that were not prespecified), separate models were fit for each covariate (condition of prediabetes vs diabetes, prescribed oral diabetes treatment, prescribed insulin, household size, social assistance, and income level), the group of allocation, an interaction term, and baseline HbA_1c_ level. Wilcoxon rank sum tests were used for patient-reported vegetable and fruit consumption, as well as β-carotene level, which had an asymmetrical distribution. A *t* test was used for serum ascorbic acid. The binary outcomes of food and financial security were analyzed using the χ^2^ test. Ordinal regression was used for general health. All secondary outcomes were compared at a 6-month follow-up. We used complete case analysis. Analysis was conducted using R statistical software for Windows (version 4.4.1; R Foundation). Statistical significance was set at *P *< .05 and all tests were 2-sided.

## Results

**Figure 1.**
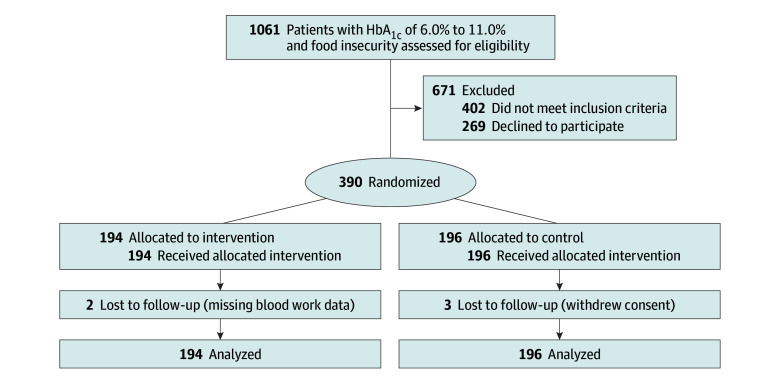
Participant Flow Diagram HbA_1c_ indicates hemoglobin A_1c_.

Between March 21, 2023, and October 3, 2024, we approached 1061 patients and enrolled 390; 194 were randomly allocated to receive the voucher and 196 to the control group (Figure 1). The characteristics of participants (mean [SD] age, 60 [13] years; 191 [49%] men, 198 [51%] women) are shown in Table 1. Overall, 81 (21%) identified as Black, 93 (24%) identified as South Asian, and 105 (27%) identified as White. Most participants had a low income (256 [66%] had an income below $30 000 per year), and 131 (34%) received social assistance, whereas 99 (25%) earned a wage. Most (317 [81%]) were prescribed oral diabetes medicines, 105 (27%) were prescribed insulin, and 72 (18%) had prediabetes. Most (201 [52%]) had hypertension, and 87 (22%) had heart disease.

**Table 1.** Demographic Characteristics of Trial Participants and Baseline Survey Responses

Characteristic	No. (%)	
	Intervention (n = 194)	Control (n = 196)
Age, mean (SD), y	60 (12)	60 (13)
Gender		
Woman	110 (57)	88 (45)
Man	84 (43)	107 (55)
Other gender	0	1 (0.5)
Indigeneity		
First Nations	6 (3)	5 (3)
Métis	3 (2)	2 (1)
None of the above	182 (94)	183 (93)
Do not know	2 (1)	6 (3)
Did not provide	1 (0.5)	1 (0.5)
Race or ethnicity		
Arab	6 (3)	3 (2)
Black	45 (23)	36 (18)
Chinese	10 (5)	6 (3)
Filipino	8 (4)	11 (6)
Japanese	2 (1)	0
Latin American	6 (3)	11 (6)
South Asian (eg, East Indian, Pakistani, Sri Lankan)	46 (24)	47 (24)
Southeast Asian (eg, Vietnamese, Cambodian, Malaysian, Laotian)	6 (3)	7 (4)
West Asian (eg, Iranian, Afghan)	4 (2)	5 (3)
White	48 (25)	57 (29)
Other^a^	14 (7)	11 (6)
Do not know	0	2 (1)
Did not provide	2 (1)	3 (2)
Household income source		
Provincial or municipal social assistance or welfare	62 (32)	69 (35)
Wages and salaries	56 (29)	43 (22)
Benefits from Canada or Quebec Pension Plan	34 (18)	30 (15)
Old age security and guaranteed income supplement	21 (11)	26 (13)
Job-related retirement pensions, superannuation and annuities	5 (3)	14 (7)
Income from self-employment	3 (2)	5 (3)
Employment insurance	2 (1)	2 (1)
Registered retirement savings plan/registered retirement income fund	2 (1)	0
Child support	0	1 (0.5)
Dividends and interest (eg, on bonds, savings)	0	1 (0.5)
Other (eg, rental income, scholarships)	8 (4)	3 (2)
None	0	1 (0.5)
Do not know	0	1 (0.5)
Did not provide	1 (0.5)	0
Household income level		
Less than $5000	2 (1)	4 (2)
$5000 to <$10 000	9 (5)	7 (4)
$10 000 to <$15 000	32 (16)	32 (16)
$15 000 to <$20 000	32 (16)	34 (17)
$20 000 to <$30 000	44 (23)	60 (31)
$30 000 to <$40 000	19 (10)	16 (8)
$40 000 to <$50 000	17 (9)	9 (5)
$50 000 to <$60 000	16 (8)	5 (3)
$60 000 to <$70 000	2 (1)	5 (3)
>$70 000	7 (4)	8 (4)
Do not know	10 (5)	13 (7)
Did not provide	4 (2)	3 (2)
No. of household members		
1	72 (37)	83 (42)
2	49 (25)	42 (21)
3	26 (13)	22 (11)
4	19 (10)	25 (13)
5	11 (6)	13 (7)
6	15 (8)	6 (3)
7	2 (1)	3 (2)
8	0	2 (1)
Highest level of education		
Some high school, no diploma	30 (15)	22 (11)
High school diploma or equivalent	39 (20)	43 (22)
Associate degree	34 (18)	22 (11)
Bachelor’s degree	40 (21)	47 (24)
Some college, no degree	18 (9)	24 (12)
Master’s degree	12 (6)	13 (7)
Trade/technical/vocational training	9 (5)	9 (5)
Professional degree	2 (1)	1 (0.5)
Doctorate degree	1 (0.5)	1 (0.5)
Other	2 (1)	0
None	6 (3)	12 (6)
Do not know	0	1 (0.5)
Did not provide	1 (0.5)	1 (0.5)
Food insecurity	188 (97)	191 (97)
Baseline diabetes condition		
Prediabetes	35 (18)	37 (19)
Diabetes treatment		
Oral medicines for diabetes	157 (81)	160 (82)
Insulin	52 (27)	53 (27)
Both oral medicines and insulin	50 (26)	44 (22)
Other conditions		
High blood pressure	102 (53)	99 (51)
Heart disease	46 (24)	41 (21)
Degenerative arthritis	46 (24)	39 (20)
Back pain	28 (14)	27 (14)
Anemia or blood disease	22 (11)	23 (12)
Lung disease	18 (9)	16 (8)
Kidney disease	18 (9)	16 (8)
Ulcer or stomach disease	18 (9)	12 (6)
Cancer	16 (8)	8 (4)
Liver disease	10 (5)	12 (6)
Rheumatoid arthritis	4 (2)	5 (3)
Did not provide	2 (1)	0

^a^ Other includes individuals who identity as First Nations or Métis; Caribbean, Indo-Caribbean, or West Indian; Jewish; or multiracial and those who identify with multiple races and/or ethnicities.

All participants in the intervention group reported receiving the voucher, and all but 1 (193 [99%]) used the vouchers. Most (150 [77%]) participants in the intervention group used all of the voucher value and 175 (90%) used more than 80% of the voucher value. Follow-up data were available for 385 participants (98.7%), with 2 (1.0%) lost to follow-up in the voucher arm and 3 (1.5%) in the control arm. Most (301 [78%]) participants completed their blood work within 1 month of the 6-month time point and the mean (SD) follow-up time was 185 (34) days (180 [28] for voucher and 190 [39] for control).

### Primary Outcome

The between-group difference in the change in HbA_1c_ from baseline to follow-up was −0.18% (95% CI, −0.41% to 0.05%; *P* = .13; Table 2). The results were similar when adjusted for baseline HbA_1c_, age, sex, household size, and condition (−0.10%; 95% CI, −0.33% to 0.12%; *P* = .35) and when additionally adjusting for site and the timing of follow-up testing (−0.11%; 95% CI −0.33% to 0.12%; *P* = .35). There was no difference in the number of patients who changed from diabetic (HbA_1c_ ≥6.5%) to normal HbA_1c_ levels (<6.0%) (6 with voucher vs 2 controls, *P* = .17), from diabetic (HbA_1c_ ≥6.5%) to prediabetics levels (HbA_1c_ between 6.0% and 6.4%) (15 vs 16, *P* = 1.0), or from prediabetic (HbA_1c_ between 6.0% and 6.4%) to normal HbA_1c_ levels (<6.0%) (3 vs 2, *P* = .68). The number of patients who converted from prediabetes (HbA_1c_ between 6.0% and 6.4%) to diabetes (HbA_1c_ ≥6.5%) was also similar (11 vs 18, *P* = .25).

**Table 2.** Mean Hemoglobin A_1c_ (HbA_1c_) Values by Group

Variable	Intervention (n = 192)	Control (n = 193)
Baseline HbA_1c_, mean (SD), %	7.5 (1.2)	7.3 (1.1)
6 mo HbA_1c_, mean (SD), %	7.4 (1.4)	7.4 (1.2)
Change HbA_1c_, mean (SD), %	−0.06 (1.25)	0.12 (1.1)
Difference in change, % (95% CI)	−0.18 (−0.41 to 0.05)	
*P* value	.13	
Difference in change % adjusted for baseline HbA_1c_, age, sex, household size, and condition (95% CI)	−0.10 (−0.33 to 0.12)	
*P* value	.35	

**Figure 2.**
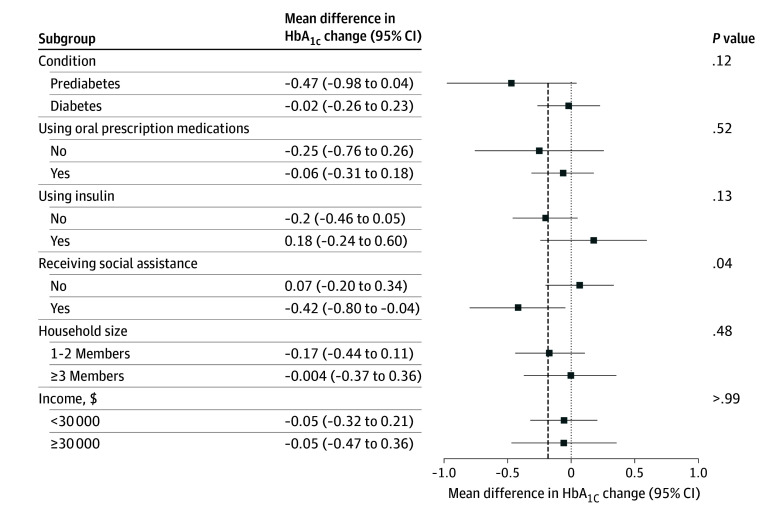
Forest Plot for Subgroup Interactions HbA_1c_ indicates hemoglobin A_1c_.

In exploratory subgroup analyses that were not prespecified, the voucher was not associated with better outcomes whether the person had prediabetes vs diabetes (−0.47%; 95% CI, −0.98% to 0.04% vs with diabetes −0.02%; 95% CI, −0.26% to 0.23%; *P* value for the interaction = .12), whether diabetes medications were prescribed or no medications (−0.25%; 95% CI, −0.76% to 0.26% vs prescribed diabetes treatments −0.06%; 95% CI, −0.31% to 0.18%; *P* = .52), whether or not insulin was prescribed (−0.20; 95% CI, −0.46 to 0.05 vs prescribed insulin 0.18; 95% CI, −0.24 to 0.60; *P* = .13), or lived in a household of 1 or 2 members (−0.17%; 95% CI, −0.44% to 0.11% vs households of 3 or more −0.004%; 95% CI, −0.37% to 0.36%; *P* = .48) (Figure 2). There was an association between voucher receipt and better outcomes and whether or not the participant received social assistance (−0.42; 95% CI, −0.80 to −0.04 vs not on social assistance 0.07; 95% CI, −0.20 to 0.34; *P* = .04).

### Secondary Outcomes

The voucher improved patient-reported consumption of vegetables (44.7% vs 21.5% two or more times per day; *P* < .001) and fruits (42.6% vs 22.7% two or more times per day; *P* < .001) (Table 3). There were no differences on objective measures of vegetable and fruit consumption, serum β-carotene (mean difference, 0.01 µmol/L; 95% CI, −0.08 to 0.06; *P* = .64) and ascorbic acid (mean difference, −0.18 µmol/L; 95% CI, −4.3 to 4.7; *P* = .94).

**Table 3.** Secondary Outcomes at 6-Month Follow-Up by Group

Variable	No. (%)		Difference (95% Cl)	*P* value	Completeness, % (No. missing)
	Intervention	Control			
Self-reported vegetable consumption, 2 or more times per day	85 (45)	37 (22)	NA	<.001	93 (28)
Self-reported fruit consumption, 2 or more times per day	81 (43)	39 (23)	NA	<.001	93 (28)
Serum β-carotene, mean (SD), µmol/L	0.33 (0.36)	0.32 (0.32)	Mean difference, 0.01 (−0.08 to 0.06)	.64	90 (40)
Serum ascorbic acid, mean (SD), µmol/L	36 (19)	37 (21)	−0.18 (−4.3 to 4.7)	.94	78 (85)
Self-reported food insecurity	142 (76)	148 (86)	Risk difference, −0.10 (−0.18 to −0.02)	.02	92 (31)
Self-reported financial insecurity	130 (72)	134 (79)	Risk difference, −0.07 (−0.16 to 0.02)	.16	89 (41)
Self-reported general health, good or very good	106 (56)	78 (45)	OR, 1.6 (1.1 to 2.3)	.02	93 (27)

Abbreviations: NA, not applicable; OR, odds ratio.

Patient-reported food insecurity was lower in the group receiving the voucher (risk difference, −0.10; 95% CI, −0.18 to −0.02; *P* = .02). Financial insecurity or the “ability to make ends meet” did not differ between groups (risk difference, −0.07; 95% CI, −0.16 to 0.02; *P* = .16).

At 6 months, patient-rated general health of good or very good was more likely in those who received the voucher (odds ratio, 1.6; 95% CI, 1.1-2.3; *P* = .02).

## Discussion

This trial found that a monthly voucher did not significantly reduce HbA_1c_ levels in people with diabetes or prediabetes experiencing food insecurity. The 95% CIs excluded a large effect on HbA_1c_. The voucher improved patient-reported vegetable consumption, fruit consumption, food security, and general health, but not financial security or objective measures associated with fruit and vegetable consumption. Exploratory subgroup analyses indicate that vouchers might be more effective in patients not prescribed medicines to control diabetes including those with prediabetes.

The voucher substantially improved self-reported vegetable and fruit consumption but not the objective measures of β-carotene, ascorbic acid, and HbA_1c_ levels. This might be explained by social desirability bias, where participants who received a voucher are more likely to report consuming healthier foods. There may also have been a real effect of the voucher on vegetable and fruit consumption that was too small or too short in duration to appreciably change objective measures. Similarly, the voucher may have reduced reported food insecurity, but not objective measures for similar reasons. Most participants who received the voucher reported food insecurity, indicating the voucher was insufficient for many.

Other trials of comparable designs in patients with type 2 diabetes who have trouble accessing healthy foods had mixed results. For example, 2 parallel-arm trials of a $10 weekly voucher for fruits and vegetables or biweekly provision of foods found improvements in HbA_1c_ levels^17,38^ and 1 parallel-arm trial of biweekly provision of groceries found no improvements in HbA_1c_ levels.^39^ Two wait-list trials, 1 of medically tailored meals and 1 of access to a pantry of healthy foods, found no effect on HbA_1c_ levels.^18,40^ In the 2 wait-list clinical trials, there were improvements in the control group during the study period.^41^

### Strengths and Limitations

The strengths of this trial include the randomized design, objective primary outcome (HbA_1c_ levels) that is associated with changes in health outcomes, the relatively large sample size compared with other trials,^17,38^ and the high rate of follow-up. The intervention paralleled usual clinical practice in that clinicians had the flexibility to adjust medical treatment, and the intervention paralleled policies in some jurisdictions because the voucher could have been used for purchases other than healthy foods. Although these 2 aspects of the intervention improved the trial’s external validity (or the extent to which it is pragmatic), they also reduce the internal validity of the trial because the voucher may not have resulted in changes that could be expected to impact the main outcome. Specifically, although participants were instructed to purchase only vegetables and fruits with the voucher, we were unable to verify what foods were purchased and consumed and in what amounts. We were also unable to determine who consumed the food purchased with the vouchers, and items may have been shared with or primarily consumed by other household members. The real value of the voucher declined after the trial was designed and funded, because the cost of food increased and most participants who received the vouchers reported food insecurity at the end of the trial.^42^ Participants had to be aware of the group of allocation and some outcomes were patient reported. Outcomes such as fruit and vegetable consumption may not be accurately self-reported by participants, and other questions regarding fruit and vegetable consumption may be more valuable, such as daily serving size. The 6-month duration of the intervention may be too short to capture slower changes in HbA_1c_ levels. Our study used the Diabetes Canada definition of prediabetes, which defines the term “prediabetes” as having an HbA_1c_ level of 6.0% to 6.4%,^43^ and this may vary from definitions used elsewhere that would include participants with an HbA_1c_ level of 5.7%.

## Conclusions

A voucher intended to improve access to healthy foods did not improve diabetes control in this trial where recipients reported more vegetable and fruit consumption. The voucher may have been insufficient in value to substantially change objective outcomes. Further studies will help to characterize and quantify effects that might, based on exploratory subgroup analyses, be larger in specific populations such as people receiving social assistance. Future studies might also evaluate the effects of different voucher sizes and the longer term changes in dietary intake and health outcomes. The effects of policy changes and interventions aimed at promoting healthy food access should be carefully evaluated in future studies while steps are taken to ensure equitable access to healthy foods.

## Notes

Supplement 1. Trial Protocol

Supplement 2. Data Sharing Statement

Supplement 3. Additional Contributions
