# Supplementary material for: Voucher for Healthy Foods and Diabetes Control: A Randomized Clinical Trial
Source: JAMA Intern Med. 2025 Oct 20;185(12):1434–41. doi: 10.1001/jamainternmed.2025.5420 (PMC12538504; doi:10.1001/jamainternmed.2025.5420)
Supplement: Supplement 1. — Trial Protocol [file jamainternmed-e255420-s001.pdf]

Trial Protocol and Statistical Analysis Plan

I. Summary of Changes to the Protocol 2
II. Original Protocol at Trial Initiation 3
III. Statistical Analysis Plan 9

I. Summary of Changes to the Protocol

Amendments to the Protocol	Approval Date
Added a third primary care site (Regent Park Community Health Centre).	2024-02-14
Sample size estimation corrected	2025-07-14
Intervention revised to remove restriction on purchases with voucher	2025-07-14

II. Original Protocol at Trial Initiation

Effect healthy food voucher on blood glucose control in people with type 2 diabetes or prediabetes: a randomized controlled trial

Background & Rationale

This study is a collaboration between a non-profit organization, Community Food Centres Canada (CFCC) and researchers.

Over 4 million Canadians (or 12.4 % of households) experience food insecurity defined as the inadequate or insecure access to food due to financial constraints. The prevalence of food insecurity has increased over the last decade (11.3 % in 2008 and 12.4 % in 2012). Food insecurity is predominantly associated with living on a low income, and numerous studies have found that when income increased, food insecurity decreased. Food insecurity is more common among social assistance recipients due to an insufficient income and this applies to individuals living in cities where the cost of living is higher. People who have inadequate income employ a variety of coping mechanisms, including cutting back on food expenditures, as one of the more elastic features of their budgets, or resorting to food charity. More than 1 million Canadians (including more than 300 000 children) visited food banks in 2018. Only a portion of those who could use a food bank actually do so, due to a variety of factors from hours of operation to the stigma that is often associated with food charity. The quality, quantity and acceptability of the food provided is often inadequate and typically includes canned foods high in sugar and salt.

In the absence of income security programs that ensure adequate means to cover basic needs or widespread programs to promote food access and potentially reduce diet-related illness in low-income communities, non-profit organizations have been taking the initiative to look at the potential of scaled subsidies to both support food access and encourage healthy eating behaviours. Community Food Centres Canada (CFCC) is a leader in the non-profit sector working on food access and food justice issues in Ontario and nationally. No trial has yet been conducted to measure the health effects of these supports. The proposed trial will provide direct evidence of the effect of healthy food provision on health outcomes among people living on low incomes in Ontario with diabetes to help inform publicly funded food programs in Ontario, and Canada more broadly.

An estimated 4.4 million Canadians have diabetes or “prediabetes” and complications including an increased risk of vascular events including myocardial infarctions and strokes and microvascular complications such as visual impairment with annual health care costs estimated above $4 billion. Standard diabetes management includes providing advice about dietary management of the disease including reducing consumption of foods with a high glycemic index such as white rice and pasta and to increase consumption of high fibre foods such as vegetables, and this type of advice has been proven in trials to reduce blood glucose levels and

proven the progression of diabetes. Individuals with a low income are less likely to adhere to dietary guidelines and are at increased risk of diabetes complications.

Food subsidies have been extensively piloted and evaluated in the United States. Facilitating access to healthy foods could free up money for the purchase of other needed goods or services. Even if a Basic Income – or other measures to ensure a sufficient income – is instituted in the future and it remains at a sufficient level indefinitely, receiving essential free goods will free up family income for other uses. There are already publicly funded programs to provide people with free tangible goods that are expected to promote health such as “baby boxes” in Finland, syringe distribution to intravenous drug users, and free food for pregnant women in Quebec. There are also programs to subsidize food for people living in remote communities in Canada.

Objectives and Specific Aims of the Project

In adults with type 2 diabetes or “prediabetes” experiencing either food insecurity or trouble making ends meet, what is the effect of a $65 food voucher monthly on hemoglobin A1c values after 6 months?

Inclusion/Exclusion Criteria

Participants will be adult (age >19 years) patients of the two participating primary care who have either “pre-diabetes” (A1c 6.0 to 6.4) or diabetes (A1c ≥ 6.5) based on bloodwork done in the last 3 months and who either score 2 or above on the six-item food insecurity instrument or who report trouble “making ends meet”. We will exclude people who live with a current study participant (to avoid contamination), people who are palliative (have a life expectancy < 6 months), people with multiple (>3) life-threatening allergies to common foods, people who require total parenteral nutrition, and people with a blood dyscrasia that interferes with hemoglobin A1c testing (anemia due to iron deficiency, B12 deficiency and folate deficiency). We will also exclude individuals with very poorly controlled diabetes (A1c > 11) as interventions other than food such as medicines may be the most important interventions. We will also exclude individuals with very poorly controlled diabetes (A1c > 11) as interventions other than food such as medicines may be the most important interventions.

Recruitment & Enrollment

Enrollment will take place within the two primary care sites in Toronto, Ontario (population 2.3 million). Notices of the study will be posted in waiting rooms and examination rooms and study brochures will also be distributed around the primary care sites. The study team will attend team meetings to make clinicians aware of the study. We will facilitate searches of electronic health records to identify potentially eligible patients and will provide clinicians with a list of

potential participants. We will ask clinicians to contact potential participants and, if they are interested in learning more about the study, clinicians will share the patient's phone number or email address with the research assistant or coordinator who will contact the patient to confirm eligibility and consent. If consent is provided, the research assistant will enter participant details in the electronic case report form that will also randomize the participant to one of the two groups. Randomization will be achieved by a web-based interface. After research personnel enter identifying details, the participant will be randomized to one of the two groups. Overall blinding is not possible because participants in the intervention group will be provided with voucher. Outcome assessors will be blinded to the group of allocation.

We expect to recruit at least 1 participant per working day and thus recruiting 390 participants may take up to 19 months. The St Michael's Academic Family Health Team is made up of six physical sites in downtown Toronto and serves approximately 45 000 patients. South Riverdale Community Health Centre is made up of six physical sites and serves approximately 10 000 patients. The collective size of the patient population across sites is approximately 55,000 and the prevalence of diabetes is 7.3 % so we estimate that there are at least 4,000 patients with diabetes and patients with diabetes typically attend appointments every 3 to 6 months and thus there are approximately 12,000 visits for diabetes each year. Based on previous waiting room surveys of patients, we estimate that at least 25 % will meet the inclusion criteria so there should be at least 1,000 patients eligible to participate. If the recruitment rate is slower than expected, we will expand the study to additional sites.

The sample size estimation is based on the power to detect a clinically important 0.4 % difference between groups in the primary outcome of the change in hemoglobin A1c. The change in hemoglobin A1c will be treated as a continuous variable. Based on previous trials, we expect a mean change in hemoglobin A1c in the control group of 0 with a standard deviation of 1.3 to 2 %.(38) To detect a 0.4 % absolute difference, a sample size of 332 to 346 is required (α 0.05, power 80 %). Assuming a 10-15 % rate of drop-out or missing data (e.g. missing hemoglobin A1c measurement), 390 participants (195 in each group) are needed. (The sample size estimation was revised to correct errors in the original protocol.)

Based on previous trials in this setting in similar populations, we expect a loss to follow up rate up to 15 %. The primary care population is relatively stable with only a small fraction (< 5 %) leaving the practice each year.

Interventions

Participants allocated to the control group will continue their usual clinical care, that includes information from clinicians about nutrition, and their usual access to food. Patients diagnosed with diabetes or who are at high risk of developing diabetes are typically offered group educational sessions about diabetes and non-pharmaceutical management approaches including diet and exercise. There is also an option to meet one-on-one with a registered dietician and a registered nurse to discuss diet and exercise. Participants allocated to the

intervention group will receive a monthly subsidy of $65 for healthy food (or $85 for participants who are members of households with more than five individuals) for fruit and vegetables every month for six months, and they will continue their usual clinical care that includes information from clinicians about nutrition. The “food prescription” voucher will be provided in the form of digital grocery cards redeemable at local retail stores - these cards were not restricted to the purchase of fresh and frozen fruits and vegetables (although that had been the earlier plan). All participants will still have access to their other regular food sources.

Design, Data Collection & Analysis

The study is an individual participant, 1:1 allocation, two-arm parallel randomized controlled trial. Allocation will be unblinded to participants and clinicians; outcome assessors and adjudicators will be blinded. Randomization will be stratified by condition: “pre-diabetes” (A1c 6.0 to 6.4) versus diabetes (A1c ≥ 6.5).

The trial design including the outcomes will be publicly registered and results reported according to CONSORT and TIDier guidance. We will make individual level participant data available when the study is complete (no identifying information about participants).

An established community guidance panel will contribute to developing the plan to implement the intervention and provide direction in study processes, interpretation and recommendations and may also identify potential additions to the study outcomes.

Study personnel will send the clinicians of participants in each group messages, through the electronic health record, at three, four and five months after randomization reminding them that the patient may be due for a hemoglobin A1c measurement. We will also contact participants by phone and email to remind them about having their hemoglobin A1c checked at 5 months.

The intervention period is six months. We will administer a survey after six months which can be completed by phone, by email, in person during a clinic visit or in person at home and will take approximately 15 minutes to complete. A subset of 20 randomly selected intervention group participants will be prompted to report all foods and beverages consumed the prior day using a validated self-administered 24-hour recall as part of this survey. In a separate subset of 20 purposefully selected intervention group participants, we will conduct interviews to capture the experience of the intervention for qualitative analysis. Prior to contacting participants to complete the survey, we will send a reminder notice by email or mail. Participants in both the intervention and control groups will be compensated $15 for completing the 6 month follow up survey. The subset of 20 participants will be compensated an additional $20 when they complete the one-on-one interview which will take approximately 20 minutes.

Hemoglobin A1c will be assessed by a review of the electronic primary care record or based on laboratory testing for study purposes if not ordered for clinical purposes.

Primary and secondary outcome measures

The primary outcome will be the hemoglobin A1c at six months adjusted for the baseline measurement (the most recent result during the 3 months prior to enrollment). Hemoglobin A1c is associated with mortality and both macrovascular and microvascular complications of diabetes. Changes in hemoglobin A1c of 0.4 % or greater are associated with clinically important reductions in at least microvascular diabetes complications. The hemoglobin A1c will be obtained through chart reviews and by accessing laboratory data with consent. If more than one hemoglobin A1c measurement is available between 3 and 6 months (even though the test is usually done every 3 months or less frequently), only the later one will be used. The primary analysis will include all participants and report A1c as a continuous measure. We will also report the change in hemoglobin A1c by condition ("prediabetes" and diabetes), the number of participants with "pre-diabetes" (A1c 6.0 to 6.4) at baseline who convert to a normal hemoglobin A1c (<6.0 %) and those who convert to diabetes (A1c ≥ 6.5), and the number of participants with diabetes at baseline (A1c ≥ 6.5) who convert to "pre-diabetes" (A1c 6.0 to 6.4) and to normal hemoglobin A1c (<6.0%).

Secondary outcome measures

Secondary outcomes will include self-reported health, self-reported food-security, self-reported financial security, carotenoid level, and self-reported fruit and vegetable consumption. Carotenoid levels will be measured during a visit at the end of the study (months 5 or 6). Serum levels of α-carotene, β-carotene, lutein, β-cryptoxanthin, and ascorbic acid correlate with self-reported vegetable and fruit consumption although the absolute value varies based on characteristics of the individual such as age and body mass index. It is thus an appropriate measure to use as a trial outcome. Self-reported health status is associated with mortality and is based on both current health and perceived health risks. The standard question is: "In general, how would you rate your health today?" Potential responses are: very good, good, moderate, bad, very bad and decline to respond. Self-reported food security will be assessed with three standard questions: "In the past 30 days, have you been concerned about having enough food for you or your family?" Potential responses are: yes, no and decline to respond. "Food you and other household members bought didn't last and there wasn't any money to get more?" Potential responses are: yes, no and decline to respond. Self-reported food security (balance): "You and other household members couldn't afford to eat balanced meals?" Potential responses are: yes, no and decline to respond. Self-reported vegetable consumption will be assessed using the standards question "How often to you eat vegetables?" Potential responses include: 0 times per day, once per day, twice per day, three times per day, four times per day, five or more times per day, or decline to answer. The same question will be applied to fruit consumption. Self-reported financial security will be assessed using the standard question "Do you have trouble making ends meet at the end of the month?"(44) Potential responses are: yes, no and decline to respond. We will ask about trade-offs between food and diabetes supplies, that is, whether participants put off purchasing food to buy diabetes supplies or vice versa (never, sometimes, often, regularly). We will track changes in medicines including changes in dose based on information available in the electronic health records. We will also

ask participants in the intervention group how often they accessed the food retailer, about their participation in educational and community building activities, about wasted food and about whether they would have wanted other foods to be provided.

We will perform a cost-effectiveness analysis from a societal perspective that considers direct and indirect costs of the intervention and direct and indirect effects including changes in healthcare utilization. We will assess the economic implications of the potential effects of the intervention including costs associated with primary care, emergency department use, and complications of diabetes based on previous studies of the association between hemoglobin A1c and health outcomes.
We will report demographic characteristics collected at the time of enrollment including age, sex, gender, education level, household income, income source, ethnicity, and medical comorbidities.

We will employ an intention to treat analysis. The primary analysis of the follow-up A1c (follow-up measurement) will be adjusted for baseline hemoglobin A1c and analysed using analysis of covariance (ANCOVA). Binary outcomes will be compared between groups using risk differences with 95% confidence intervals, and chi-squared tests to derive two-sided p-values. Continuous outcomes will be compared between groups using difference in means with 95% confidence intervals, and t-tests to derive two-sided p values. Count outcomes will be analysed using rate ratios from quasi-Poisson models with 95% confidence intervals and two-sided p-values, which will be adjusted for baseline values whenever available. Ordinal outcomes will be analyzed using ordinal regression.

Randomization will be stratified by site. We will report both the unadjusted analysis for the primary outcome and the analysis adjusted for age, sex, whether or not prescribed diabetes oral medicines at baseline, and whether or not prescribed insulin at baseline.

A subset of 20 randomly selected intervention group participants will be prompted to report all foods and beverages consumed the prior day using a validated self-administered 24-hour recall (ASA24 http://riskfactor.cancer.gov/tools/instruments/asa24/). In a separate subset of 20 purposefully selected intervention group participants, we will conduct one-on-one interviews over the phone to capture the experience of the intervention for qualitative analysis. If participants agree, the interviews will be audio recorded. The transcripts will then be coded and analyzed by two research coordinators. The results of these studies will be reported together.

We will present results by diagnosis (diabetes versus “prediabetes”), age, sex, gender and income level.

III. Statistical Analysis Plan

Food Prescription Trial: Statistical Analysis Plan
9 March 2025

1. Background

Over 4 million Canadians (or 12.4 % of households) experience food insecurity defined as the inadequate or insecure access to food due to financial constraints (1). Most likely to experience food insecurity are people who live on low incomes, including those who receive social assistance in cities with high costs of living (1–3). Food insecurity is associated with increased risk for type 2 diabetes (4), as well as poor glycemic control and diabetes distress for those already diagnosed (5–7). Individuals with a low income and diabetes or prediabetes are less likely to adhere to dietary management guidelines and are at an increased risk of developing complications (8–10). Food insecurity is correlated with reduced diet quality and lower consumption of healthy foods in adults with diabetes (11). Facilitating access to healthy foods could free up money for the purchase of other needed goods or services.

2. Trial

The Food Prescription study was a randomized-controlled trial that measured the effects of a monthly food voucher on diabetes-related health outcomes. Participants were randomized to either to the control group, where they would continue to receive usual clinical care, or to the intervention group, where they received a monthly subsidy of $65 or $85 for healthy food every month for six months on top of their usual clinical care. Outcomes were measured at 6 months. Whether participants received a $65 or $85 monthly voucher was determined by the number of individuals in the household, most participants received $65 monthly and those living in households with more than 5 members received $85 monthly.

2.1 Primary Research Question

In adults with type 2 diabetes or “prediabetes” experiencing either food insecurity or trouble making ends meet, what is the effect of a $65 (or $85) monthly food voucher for six months on the change in hemoglobin A1c values?

2.2 Trial Design

We conducted an open label, parallel two-arm, 1:1 allocation randomized controlled trial. Randomization is stratified by condition: “pre-diabetes” (A1c 6.0 to 6.4) versus diabetes (A1c ≥ 6.5).

3. Objectives

3.1 The primary objective of this trial is to test the following hypothesis:

1. A monthly subsidy of $65 (or $85) for healthy food every month for six months is superior to usual care in decreasing HbA1c levels after 6 months in adults with type 2 diabetes or “prediabetes” experiencing food insecurity or trouble making ends meet

3.2 The secondary objectives of this trial are to test the following hypotheses:

1. A monthly subsidy of $65 (or $85) for healthy food every month for six months is superior to usual care in its effect on beta-carotene in adults with type 2 diabetes or “prediabetes” experiencing food insecurity or trouble making ends meet

2. A monthly subsidy of $65 (or $85) for healthy food every month for six months is superior to usual care in its effect on ascorbic acid in adults with type 2 diabetes or “prediabetes” experiencing food insecurity or trouble making ends meet
3. A monthly subsidy of $65 (or $85) for healthy food every month for six months is superior to usual care in its effect on self-reported health status in adults with type 2 diabetes or “prediabetes” experiencing food insecurity or trouble making ends meet
4. A monthly subsidy of $65 (or $85) for healthy food every month for six months is superior to usual care in its effect on self-reported financial security in adults with type 2 diabetes or “prediabetes” experiencing food insecurity or trouble making ends meet
5. A monthly subsidy of $65 (or $85) for healthy food every month for six months is superior to usual care in its effect on food security status in adults with type 2 diabetes or “prediabetes” experiencing food insecurity or trouble making ends meet
6. A monthly subsidy of $65 (or $85) for healthy food every month for six months is superior to usual care in its effect on self-reported vegetable consumption in adults with type 2 diabetes or “prediabetes” experiencing food insecurity or trouble making ends meet
7. A monthly subsidy of $65 (or $85) for healthy food every month for six months is superior to usual care in its effect on self-reported fruit consumption in adults with type 2 diabetes or “prediabetes” experiencing food insecurity or trouble making ends meet

4. Study population

4.1 Participant flow
A CONSORT patient flow diagram will be drawn following the CONSORT 2010 standards (http://www.consort-statement.org/consort-2010). The flow chart will consider specifically:

- N assessed for eligibility
- N not included in trial (with reasons)
- N randomized
- N allocated to intervention/control
  - N receiving allocated intervention
  - N not receiving allocated intervention (with reasons)
- N followed up at 6 months (with reasons for termination)
- N analyzed
  - N excluded from primary analysis (with reasons)

4.2. Intention-to-treat (ITT) population
The ITT population consists of all randomized participants. Participants will be analysed regardless of whether they actually received the allocated intervention or not or any other protocol deviations in the group they were originally allocated to.

4.3 *Complete-cases (CC) population*
The CC population consists of all randomized participants who have completed the assessment for the primary outcome data at follow-up.

5. Data Management

5.1. Data export

Trial data will be provided by the Applied Health Research Centre and will be imported from the electronic data capturing system into R by the trial statistician for data preparation, validation and analysis.

5.2. Data validation

All variables used in the analysis, including the derived variables, will be checked for missing values, outliers, and inconsistencies and queried.

5.3 Data preparation

The primary outcome of HbA1c at 6 months will be assessed via laboratory testing for study purposes or review of the electronic primary care record. This is a continuous variable. The primary outcome will be adjusted for HbA1c at baseline, which will be assessed via review of the electronic primary care record. This is a continuous variable.

Serum β-carotene and ascorbic acid levels at 6 months will be assessed via laboratory testing for study purposes.

Data on self-reported food security, self-reported financial security, and self-reported vegetable and fruit consumption will be collected through surveys administered online, over the phone, or in-person at 6 months. Baseline data on self-reported food security and baseline self-reported vegetable and fruit consumption were collected at through surveys conducted over the phone or in-person at the recruitment visit.

Self-reported health status will be measured on a 5-point likert scale, where participants were asked the standard question at follow-up "In general, how would you rate your health today?" Potential responses are: very good, good, moderate, bad, very bad and decline to respond.

Self-reported food security will be assessed using 6 standard and validated items adapted from the Canadian Community Health Survey (Household Food Security Survey Module), applied to the last 6 months (12). The instrument assesses worry about food running out due to financial constraints, food not lasing, and the inability to afford balanced meals using the following questions:

1. The food that you and other household members bought just didn't last, and there wasn't any money to get more. Was that often true, sometimes true, or never true in the past 6 months? Potential responses are: Often true, Sometimes true, Never true, Don't know/Refuse to answer
2. You and other household members couldn't afford to eat balanced meals. Was that often true, sometimes true, or never true in the past 6 months? Potential responses are: Often true, Sometimes true, Never true, Don't know/Refuse to answer
3. In the past 6 months, did you or other adults in your household ever cut the size of your meals or skip meals because there wasn't enough money for food? Potential responses are: Yes, No, Don't know/Refuse to answer
    a. (If answered Yes to question 3) How often did this happen? Potential responses are: Almost every month, Some months but not every month, Only 1 or 2 months, Don't know/Refuse to answer
4. In the past 6 months, did you (personally) ever eat less than you felt you should because there wasn't enough money to buy food

5. In the past 6 months, were you (personally) ever hungry but didn't eat because you couldn't afford enough food? Potential responses are: Yes, No, Don't know/Refuse to

Participants can answer affirmatively to 0 to 6 of the questions. We will assess food security status as a binary variable, where less than 2 affirmative responses indicates "food secure" and 2 or more affirmative responses indicates more "food insecure." This is based on Health Canada's approach to interpreting the data from the Household Food Security Survey Module (13). We will also examine food security as a raw score between 0 to 6 (based on the number of affirmatively answered questions) as a sensitivity analysis.

Self-reported financial security is a categorical variable where participants were asked the standard question at follow-up "Do you have trouble making ends meet at the end of the month?" Potential responses were: yes, no and decline to respond.

Self-reported vegetable consumption and self-reported fruit consumption are both categorical variables, measured at both baseline and 6 months. Participants were asked "How often to you eat vegetables?" and "How often do you eat fruits?" Potential responses included: 0 times per day, once per day, twice per day, three times per day, four times per day, five or more times per day, or decline to answer.

6. Outcomes

6.1 Baseline characteristics

The following baseline characteristics will be summarized descriptively by group:

- Age
- Sex
- Gender
- Ethnicity
- Household income
- Income source
- Level of education
- Household size
- Prescribed oral prescription medications
- Prescribed insulin

6.2 Primary Outcome

The primary outcome is the change in hemoglobin A1c from baseline to follow up at six months. This is a continuous variable.

6.3 Secondary Outcomes:

1. Serum β-carotene levels (a continuous variable, expressed in units of µmol/L)
2. Serum ascorbic acid levels (a continuous variable, expressed in units of µmol/L)
3. Self-reported health status (an ordinal variable with the following categories: very good, good, moderate, bad, and very bad)
4. Self-reported financial security (a binary variable with the following categories: yes and no)
5. Self-reported food security (an ordinal variable with values between 0 to 6 and a binary variable with the following categories: food secure and food insecure)

6. Self-reported vegetable consumption (a count variable with values between 0 to 5 times per day)
7. Self-reported fruit consumption (a count variable with values between 0 to 5 times per day)

7. Sample size

The sample size was estimated based on the power to detect a clinically important 0.04 difference between groups in the primary outcome of the change in hemoglobin A1c. Based on previous trials, we expect a mean change in hemoglobin A1c in the control group of 0, with a standard deviation of 0.013 (1.3%). To detect a 0.004 (0.4%) absolute difference, a sample size of 332 (166 in each group) is required (α 0.05, power 80%). Assuming a 15% rate of drop-out or missing data (e.g. missing hemoglobin A1c measurement), 390 participants (195 in each group) are needed. A total of 390 participants were recruited, with 194 allocated to the intervention group and 196 allocated to the control group.

8. Statistical Analysis

8.1 Participant baseline characteristics

Participant characteristics at baseline will be summarized by trial arm. Continuous variables will be summarized using mean and standard deviation, or median and inter-quartile range if data do not have a symmetric distribution. Categorical variables will be summarized with counts and percentages. No statistical comparisons of participant characteristics at baseline will be performed between groups.

8.2 Analysis of primary outcome

Hemoglobin A1c will be compared between the intervention and control arm using analysis of covariance (ANCOVA). The covariates will be age, sex, household size, and condition (diabetes versus prediabetes). We will present results of the adjusted and unadjusted models. We will also describe for each group (intervention and control) the baseline, follow-up (6 month) and change (follow-up minus baseline) A1c using mean (with standard deviation) and median (with interquartile range).

In a sub-group analyses, we will assess whether there is a significant interaction between the mean change in hemoglobin A1c and (1) age, (2) sex, (3) condition ("prediabetes" and diabetes), (4) household size, (5) whether prescribed oral diabetes treatment (6) whether prescribed insulin.

We will also use chi-square tests to compare the proportion of participants with "pre-diabetes" (A1c 6.0 to 6.4) at baseline who convert to a normal hemoglobin A1c (<6.0 %) and those who convert to diabetes (A1c ≥ 6.5), and the number of participants with diabetes at baseline (A1c ≥ 6.5) who convert to "pre-diabetes" (A1c 6.0 to 6.4) and to normal hemoglobin A1c (<6.0%).

For the primary analysis, a *p*-value of <0.05 will be sufficient to reject the null hypothesis of no difference.

We will address missing outcome data using methods appropriate to their pattern which may include inverse probability weighted analysis. We will address missing baseline characteristic data using methods appropriate to their pattern which may include multiple imputation.

8.3 Analysis of secondary outcomes

The continuous outcomes of serum β-carotene and ascorbic acid levels will be compared between groups using difference in means with 95% confidence intervals, and t-tests will be used to derive two-sided *p*-values. Alternative non-parametric tests will be used if the data does not meet the assumptions of t-tests and these may include the Wilcoxon Rank Sum test.

The binary outcomes of self-reported financial security and food security status will be compared between groups using risk differences with 95% confidence intervals, and chi-squared tests to derive two-sided p-values.

The count outcomes of self-reported vegetable and fruit consumption, will be compared between groups using the Wilcoxon rank sum test, where a *p*-value of <0.05 will be sufficient to reject the null hypothesis of no difference.

For the ordinal outcomes of self-reported health-status and food-security score ordinal regression will be used using odds ratios with 95% confidence intervals.

References

1. Statistics Canada. Household food insecurity, 2017/2018 [Internet]. 2020 [cited 2021 Sep 9]. Available from: https://www150.statcan.gc.ca/n1/pub/82-625-x/2020001/article/00001-eng.htm

2. Tarasuk V, Li N, Dachner N, Mitchell A. Household Food Insecurity in Ontario during a Period of Poverty Reduction, 2005–2014. Canadian Public Policy. 2019 Mar 19;45(1):93–104.

3. Dachner N, Tarasuk V. Tackling household food insecurity: An essential goal of a national food policy. Canadian Food Studies / La Revue canadienne des études sur l'alimentation. 2018 Sep 30;5(3):230–47.

4. Tait CA, L'Abbé MR, Smith PM, Rosella LC. The association between food insecurity and incident type 2 diabetes in Canada: A population-based cohort study. Meyre D, editor. PLoS ONE. 2018 May 23;13(5):e0195962.

5. Berkowitz SA, Baggett TP, Wexler DJ, Huskey KW, Wee CC. Food Insecurity and Metabolic Control Among U.S. Adults With Diabetes. Diabetes Care. 2013 Oct 1;36(10):3093–9.

6. Bawadi HA, Ammari F, Abu-Jamous D, Khader YS, Bataineh S, Tayyem RF. Food insecurity is related to glycemic control deterioration in patients with type 2 diabetes. Clinical Nutrition. 2012 Apr;31(2):250–4.

7. Silverman J, Krieger J, Kiefer M, Hebert P, Robinson J, Nelson K. The Relationship Between Food Insecurity and Depression, Diabetes Distress and Medication Adherence Among Low-Income Patients with Poorly-Controlled Diabetes. J GEN INTERN MED. 2015 Oct;30(10):1476–80.

8. Yaghoubi M, Mansell K, Vatanparast H, Steeves M, Zeng W, Farag M. Prevalence of Type 1 and Type 2 Diabetes-Related Complications and Their Association With Determinants Identified in Canada's Survey on Living With Chronic Diseases—Diabetes Component. Canadian journal of diabetes. 2020;44(4):304-311.e3.

9. Hosseini Z, Whiting SJ, Vatanparast H. Type 2 diabetes prevalence among Canadian adults — dietary habits and sociodemographic risk factors. Applied physiology, nutrition, and metabolism. 2019;44(10):1099–104.

10. Gagné T, Veenstra G. Inequalities in Hypertension and Diabetes in Canada: Intersections between Racial Identity, Gender, and Income. Ethnicity & disease. 2017;27(4):371–8.

11. Fu P, Wen J, Duan X, Hu X, Chen F, Yuan P. Association between adult food insecurity and mortality among adults aged 20–79 years with diabetes: A population-based retrospective cohort study. Diabet Med. 2024 Apr;41(4):e15268.

12. Health Canada. Canadian Community Health Survey Cycle 2.2, Nutrition (2004): Income-Related Household Food Security in Canada. 2007. Report No.: 4696.

13. Health Canada. Determining food security status [Internet]. 2020 [cited 2025 Mar 19]. Available from: https://www.canada.ca/en/health-canada/services/food-nutrition/food-nutrition-surveillance/health-nutrition-surveys/canadian-community-health-survey-cchs/household-food-insecurity-canada-overview/determining-food-security-status-food-nutrition-surveillance-health-canada.html
