# Supplementary material for: Voucher for Healthy Foods and Diabetes Control: A Randomized Clinical Trial
Source: JAMA Intern Med. 2025 Oct 20;185(12):1434–41. doi: 10.1001/jamainternmed.2025.5420 (PMC12538504; doi:10.1001/jamainternmed.2025.5420)
Supplement: Supplement 2. — Data Sharing Statement [file jamainternmed-e255420-s002.pdf]

Data Sharing Statement

Persaud. Voucher for Healthy Foods and Diabetes Control. *JAMA Intern Med*. Published October 20, 2025. doi:10.1001/jamainternmed.2025.5420

Data
Additional Information: ClinicalTrials.gov Identifier: NCT05776420, registered Mar 16, 2023 (https://clinicaltrials.gov/study/NCT05776420?cond=diabetes&term=Persaud&rank=1)
Data available: Yes
Data types: Deidentified participant data
How to access data: The data will be made available upon reasonable request to the corresponding author (nav.persaud@utoronto.ca).
When available: With publication

Supporting Documents
Document types: None

Additional Information
Who can access the data: Anyone requesting the data.
Types of analyses: For any purpose.
Mechanisms of data availability: With Investigator support.
