# Supplementary material for: Voucher for Healthy Foods and Diabetes Control: A Randomized Clinical Trial
Source: JAMA Intern Med. 2025 Oct 20;185(12):1434–41. doi: 10.1001/jamainternmed.2025.5420 (PMC12538504; doi:10.1001/jamainternmed.2025.5420)
Supplement: Supplement 3. — Additional Contributions [file jamainternmed-e255420-s003.pdf]

Additional Acknowledgements

Mo Alhaj	St. Michael's Hospital	Quality Improvement Decision Support Specialist
Karin Bogad	St. Michael's Hospital	RN at 80 Bond site
Lauren Brouhard-Chuck	St. Michael's Hospital	Social worker at Wellesley-St James site
Jacqueline	St. Michael's Hospital	Senior Clinical Program Director
Haysook Choi	St. Michael's Hospital	Diabetes RN at 80 bond site
Margaret Kohut	St. Michael's Hospital	Admin/Clerical Coordinator at Wellesley-St James site
Marija Kundacina	St. Michael's Hospital	Admin/Clerical Coordinator at 80 Bond site
Paula Pereira	St. Michael's Hospital	Admin/Clerical Coordinator at Sumac Creek site
Smitha Pradhan	St. Michael's Hospital	Dietitian at 80 Bond site
Roy Paulo	St. Michael's Hospital	RN at 80 Bond site
Rae Anne Roman	St. Michael's Hospital	RN at 61 Queen site
Carri-Lynn Wannamaker	St. Michael's Hospital	Admin/Clerical Coordinator at 61 Queen site
Gordon Arbess	St. Michael's Hospital	Clinician
Gary Bloch	St. Michael's Hospital	Clinician
Paul Das	St. Michael's Hospital	Clinician
Mary Beth Derocher	St. Michael's Hospital	Clinician
Kathryn Dorman	St. Michael's Hospital	Clinician
Kathleen Doukas	St. Michael's Hospital	Clinician
Allison Farber	St. Michael's Hospital	Clinician
Amy Freedman	St. Michael's Hospital	Clinician
Graham Gaylord	St. Michael's Hospital	Clinician
Abbas Ghavam-Rassoul	St. Michael's Hospital	Clinician
Rajesh Girdhari	St. Michael's Hospital	Clinician
Rick Glazier	St. Michael's Hospital	Clinician
Ritika Goel	St. Michael's Hospital	Clinician
Laurie Green	St. Michael's Hospital	Clinician
Samantha Green	St. Michael's Hospital	Clinician
Curtis Handford	St. Michael's Hospital	Clinician
Margaret Hess	St. Michael's Hospital	Clinician
Charlotte Hunter	St. Michael's Hospital	Clinician
Karl Iglar	St. Michael's Hospital	Clinician
Gwen Jansz	St. Michael's Hospital	Clinician
Emma Jeavons	St. Michael's Hospital	Clinician
Liana Kaufman	St. Michael's Hospital	Clinician
Tara Kiran	St. Michael's Hospital	Clinician

Holly Knowles	St. Michael's Hospital	Clinician
Bruce Kwok	St. Michael's Hospital	Clinician
Margarita Lam-Antoniades	St. Michael's Hospital	Clinician
Renata Leong	St. Michael's Hospital	Clinician
Fok-Han Leung	St. Michael's Hospital	Clinician
Erin Lurie	St. Michael's Hospital	Clinician
Jennifer McCabe	St. Michael's Hospital	Clinician
Sharon Mintz	St. Michael's Hospital	Clinician
Judith Peranson	St. Michael's Hospital	Clinician
Julia Rackal	St. Michael's Hospital	Clinician
Nasreen Ramji	St. Michael's Hospital	Clinician
Noor Ramji	St. Michael's Hospital	Clinician
Danyaal Raza	St. Michael's Hospital	Clinician
Genevieve Rochon-Terry	St. Michael's Hospital	Clinician
Claire Rollans	St. Michael's Hospital	Clinician
Esther Rosenthal	St. Michael's Hospital	Clinician
Caroline Ruderman	St. Michael's Hospital	Clinician
Manjot Sarao	St. Michael's Hospital	Clinician
Susan Shepherd	St. Michael's Hospital	Clinician
Rami Shoucri	St. Michael's Hospital	Clinician
Anushi Sivarajah	St. Michael's Hospital	Clinician
Ann Stewart	St. Michael's Hospital	Clinician
Karen Swirsky	St. Michael's Hospital	Clinician
Joshua Tepper	St. Michael's Hospital	Clinician
Phillip Tsang	St. Michael's Hospital	Clinician
Barbara Vari	St. Michael's Hospital	Clinician
Priya Vasa	St. Michael's Hospital	Clinician
Zoe Von Aesch	St. Michael's Hospital	Clinician
Cheng Tao Wang	St. Michael's Hospital	Clinician
William Watson	St. Michael's Hospital	Clinician
Thea Weisdorf	St. Michael's Hospital	Clinician
Karen Weyman	St. Michael's Hospital	Clinician
Sheila Wijayasinghe	St. Michael's Hospital	Clinician
Patricia Windrim	St. Michael's Hospital	Clinician
